# A Review of MEMS Vibrating Gyroscopes and Their Reliability Issues in Harsh Environments

**DOI:** 10.3390/s22197405

**Published:** 2022-09-29

**Authors:** Waqas Amin Gill, Ian Howard, Ilyas Mazhar, Kristoffer McKee

**Affiliations:** Department of Mechanical Engineering, Curtin University, Perth, WA 6845, Australia

**Keywords:** MEMS vibrating gyroscope, tuning fork, gimbal, vibrating ring, mode mismatch, frequency modulated, rate integrated, space applications

## Abstract

Micro-electromechanical systems (MEMS) vibrating gyroscopes have gained a lot of attention over the last two decades because of their low power consumption, easy integration, and low fabrication cost. The usage of the gyroscope equipped with an inertial measurement unit has increased tremendously, with applications ranging from household devices to smart electronics to military equipment. However, reliability issues are still a concern when operating this inertial sensor in harsh environments, such as to control the movement and alignment of mini-satellites in space, tracking firefighters at an elevated temperature, and assisting aircraft navigation in gusty turbulent air. This review paper focuses on the key fundamentals of the MEMS vibrating gyroscopes, first discussing popular designs including the tuning fork, gimbal, vibrating ring, and multi-axis gyroscopes. It further investigates how bias stability, angle random walk, scale factor, and other performance parameters are affected in harsh environments and then discusses the reliability issues of the gyroscopes.

## 1. Introduction

The gyroscope is an inertial sensor that is used for the measurement or control of the orientation and rotational velocity of a body. In the early 17th century, people occasionally used spinning mass objects for navigation purposes. The spinning mass gyroscope concept was first developed by French scientist Jean Bernard Leon Foucault in 1852 [1]. In the late 18th century, the usage of the gyroscope was extended to ship navigation at sea. At the beginning of the 20th century, the traditional spinning mass gyroscope started to be used in aircraft [2]. In the 1960s, the concept of optical lasers for gyroscopes was introduced, which provided higher precision and better sensitivity and brought a tremendous leap forward for aerospace and military applications [3]. However, the costs associated with optical gyroscopes were quite high, and this provided motivation for the development of micro-electromechanical systems (MEMS) vibrating gyroscopes. Over the past few decades, a large number of MEMS gyroscopic technologies have been developed with high sensitivity, high scale factor, and reduced fabrication costs [4].

Nowadays, in our daily life routine, smart devices are commonly used for tracking and their navigation capability requires global positioning systems, such as mobile phones, smartwatches, and vehicles. The navigation systems comprise inertial measurement units (IMU) [5], which are installed in the smart electronic devices [6]. The IMU typically consists of multiple inertial sensors, including a gyroscope, accelerometer, and magnetometers. All of these sensors work from different scientific principles: the gyroscope is a rotational motion inertial sensor that detects the change of position when rotation occurs, the accelerometer is a translational motion sensor that detects linear acceleration [7], and the magnetometer gives guidance in the coordinate system [8].

The usage of the MEMS gyroscope has increased enormously over the last 20 years. These sensors have been extensively used in smart devices, automotive industries, household applications, aerospace, military applications, and so on [9,10]. The research on the MEMS vibratory gyroscopes started gaining maturity and moved towards practical designs at the start of the 21st century. In the early stages, only a few research groups tended to research in this area. However, at the beginning of the 2000s, more research groups showed interest and developed a variety of designs for MEMS vibrating gyroscopes [11]. The gyroscope’s sensitivity and performance degrade when it is exposed to an unwanted atmosphere. Some of the prominent issues that deteriorate the stability and reliability of MEMS for gyroscopes range from microfabrication process stability (beam stiffness, material properties, and critical dimension losses) to exposure to harsh environments (space, elevated temperature, radiation) and external vibrations. This review paper discusses: (1) the basic operations of the MEMS vibratory gyroscopes, (2) the development of the various types of gyroscopes, and (3) the potential reliability issues with the MEMS vibrating gyroscopes at elevated temperatures, high external vibrations, and some microfabrication processing errors.

## 2. Fundamentals of Vibrating Gyroscopes

The basic principle of the MEMS vibrating gyroscope consists of two operational modes of vibration: one is the driving mode that provides a constant and continuous vibration along the driving axis, and the other is the sensing mode that detects the movement of the vibration along the sensing axis when the external rotation is provided.

### 2.1. Basic Architecture

**Figure 1:**
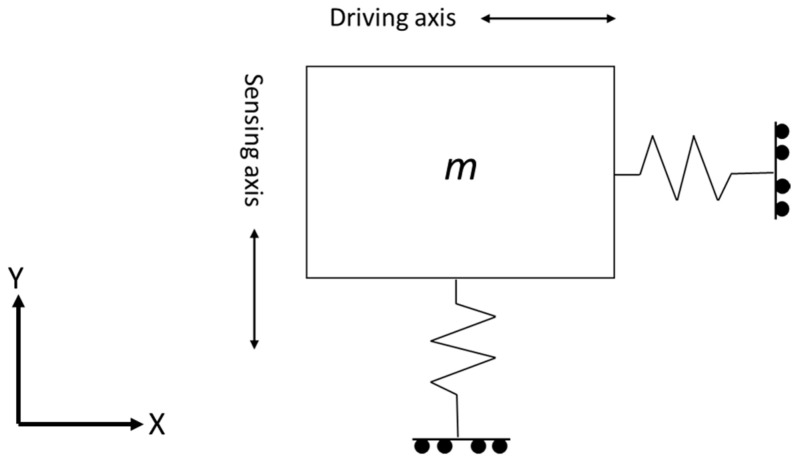
Simple depiction of a proof mass vibrating system.

Most MEMS vibrating gyroscopes have proof masses “*m*” with two orthogonal degrees of freedom, vertical and horizontal. When the gyroscope continuously vibrates at a resonant frequency in one direction, the oscillation remains absent in the orthogonal direction until the external rotation has occurred in the gyroscope. When the external rotation has occurred, this rotation triggers the energy of the first vibration to shift towards the orthogonal direction due to the Coriolis effect [12]. The simplest schematic diagram of the two degrees of freedom mass vibrating system is shown in Figure 1.

The simplest two degrees of freedom equations of motion for MEMS vibrating gyroscopes and the equation of the Coriolis force are given below.

(1) $$m\frac{d^{2}x}{dt^{2}}+c\;\frac{dx}{dt}+kx=F_{D}+2m\Omega \frac{dy}{dt}$$

The response of the sensing mode is generally very small in magnitude compared to the driving mode response, so the Coriolis force $2m\Omega \frac{dy}{dt}$ will become negligible, and Equation (1) can be written as below.

(2) $$m\frac{d^{2}x}{dt^{2}}+c\;\frac{dx}{dt}+kx=F_{D}$$

(3) $$m\frac{d^{2}y}{dt^{2}}+c\;\frac{dy}{dt}+ky=F_{S}-F_{C}$$

(4) $$F_{C}=-2m\Omega \frac{dx}{dt}$$

where $\;F_{D},F_{S},\;\mathrm{and}\;F_{C}$ are the driving, sensing, and Coriolis forces, respectively, *m* is the inertial mass, *x* is the displacement of the driving motion, *y* is the displacement of the sensing motion, *c* is the damping coefficient, *k* is the stiffness constant, and Ω is the external rotation rate.

**Figure 2:**
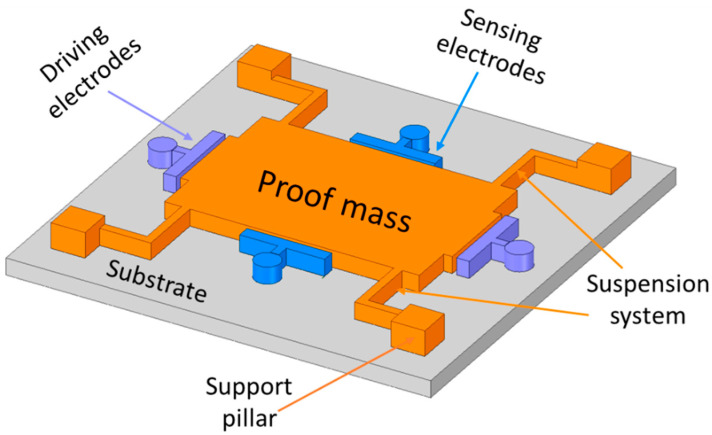
Schematic architecture of a single proof mass vibrating gyroscope.

The simplest structure of a MEMS vibratory gyroscope is shown in Figure 2, with a proof mass placed above the substrate, driving and sensing electrodes, and a suspension support beam system with four fixed support pillars [13]. The driving system comprises driving electrodes that provide an oscillation with a certain amplitude, and the sensing system, which is composed of a Coriolis force detector. The proof mass becomes an oscillator when the driving electrodes provide a constant and continuous momentum along the driving axis direction. The same proof mass upon external rotation changes its oscillation movement to the orthogonal direction of the driving axis. The sensing electrodes on the orthogonal axis detect the Coriolis force that is produced by the mixture of driving momentum and external rotation.

### 2.2. Drive-Mode Operation

**Figure 3:**
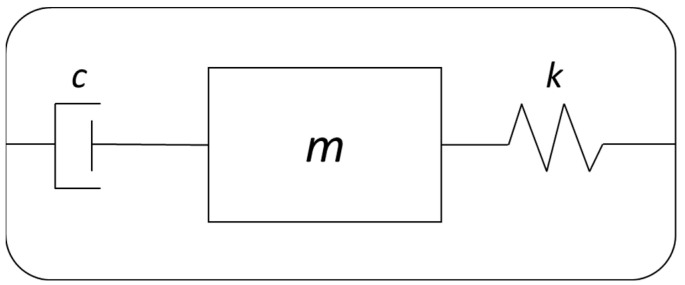
Typical one degree of freedom resonator.

Many of the MEMS vibrating gyroscopes comprise the combination of proof masses with a spring system to form a one degree of freedom resonator scheme, as shown in Figure 3.

The equation of motion for one degree of freedom is given below. Here, “*m*” is the proof mass, “*c*” is the damping constant, and “*k*” is the stiffness constant.

(5) $$m\frac{d^{2}x}{dt^{2}}+c\frac{dx}{dt}+kx=F$$

(6) $$\zeta =\frac{c}{c_{c}}=\frac{c}{2\sqrt{km}}$$

(7) $$\omega_{n}=\sqrt{\frac{k}{m}}$$

Substituting into the equation the natural frequency $“\omega_{n}”$ and the damping factor “ζ”, which is the ratio of damping to critical damping, the equation of motion for one degree of freedom system becomes:

(8) $$\frac{d^{2}x}{dt^{2}}+2\omega_{n}\zeta \frac{dx}{dt}+\omega_{n}{}^{2}x=\frac{F}{m}$$

The Coriolis force relies on the law of conservation of momentum. A vibrating gyroscope requires sensitive mechanical parts suspended on the substrate that produces momentum in a certain direction. In MEMS vibratory gyroscopes, an oscillator system must be included with the driving electrodes, generating the harmonic perturbation and maintaining this momentum along the driving axis. The equation of motion for the driving operation is given as:

(9) $$m_{D}\frac{d^{2}x}{dt^{2}}+c_{D}\frac{dx}{dt}+k_{D}x=F_{D}\sin\omega t$$

where $m_{D}$ is the driving mass, $c_{D}$ is a driving damping constant, and $k_{D}$ is the driving spring stiffness constant for the one degree of freedom resonator.

The vibrating structure needs to be excited harmonically with the force *F* = *F*_0_ sin *ωt*. The quality factor of the driving mode $Q_{D}$ and driving resonant frequency $\omega_{D}$ defines the amplitude response [14] for the harmonic excitation, and is written as in Equation (10).

(10) $$x=x_{0}\;\sin\left(\omega t+\phi \right)$$

(11) $$x_{0}=\frac{F_{0}}{k\sqrt{{\left[1-{\left(\frac{\omega }{\omega_{D}}\right)}^{2}\right]}^{2}+{\left[\frac{\omega }{Q_{D}\omega_{D}}\right]}^{2}}}$$

(12) $$\omega_{D}=\sqrt{\frac{k_{D}}{m_{D}}}$$

(13) $$Q_{D}=\frac{m_{D}\omega_{D}}{c_{D}}$$

The quality factor and scale factor are important parameters to describe the efficiency and performance of the gyroscope and they are crucial to sustaining the stable driving resonant amplitude, as the quality factor and scale factor are directly proportional to the given amplitude. The driving mode resonant frequency amplitude can be described as in Equation (14).

(14) $$x_{0}=\frac{Q_{D}F_{D}}{m_{D}\omega^{2}{}_{D}}$$

### 2.3. Sense-Mode Operation

The sense-mode operation depends on the Coriolis effect. The Coriolis force $F_{C}$ needs a drive-mode operation that results in the proof mass continuously vibrating at the given resonant frequency via driving electrodes along the driving axis. When the gyroscope is subjected to rotation, then the developed Coriolis force excites the sensing-mode system as given in Equation (4).

Consider that the sensing-mode system also has one degree of freedom resonator, similar to the driving-mode system. To understand the Coriolis force, it is assumed that the drive-mode system vibrates at a driving resonant frequency with a stable driving amplitude $\;x_{0}$. The drive motion amplitude is given as $x=x_{0}\sin\left(\omega t+\phi \right).\;$ The Coriolis force equation becomes:

(15) $$F_{c}=-2m_{C}\Omega x_{0}\omega_{D}\cos\left(\omega_{D}t+\phi_{D}\right)$$

In a single mass system, to achieve the constant scale factor, the amplitude of the Coriolis force is directly proportional to the driving amplitude. The equation of motion for the one degree of freedom resonator for the sensing-mode system is given below. Here, $m_{S}$ is the sensing mass, $m_{C}$ is the Coriolis mass, and $m_{D}$ is the driving mass. All of the masses are equal in the one degree of freedom resonator.

(16) $$m_{S}\frac{d^{2}y}{dt^{2}}+c_{S}\frac{dy}{dt}+k_{S}y=-2m_{C}x_{0}\omega_{D}\Omega \cos\left(\omega_{D}t+\phi_{D}\right)$$

The quality factor of the sensing mode $Q_{S}$ and sensing resonant frequency $\omega_{S}$ defines the sensing mode amplitude, which is written below.

(17) $$y_{0}=\left(\frac{\Omega m_{C}\omega_{D}}{m_{S}\omega^{2}{}_{S}}\right)\frac{F_{0}}{k\sqrt{{\left[1-{\left(\frac{\omega_{D}}{\omega_{S}}\right)}^{2}\right]}^{2}+{\left[\frac{\omega_{D}}{Q_{S}\omega_{S}}\right]}^{2}}}$$

(18) $$\omega_{S}=\sqrt{\frac{k_{S}}{m_{S}}}$$

(19) $$Q_{S}=\frac{m_{S}\omega_{S}}{c_{S}}$$

The best way to obtain the highest gain response in the sensing mode is by matching the driving and sensing resonant frequencies. The sensing mode resonant frequency amplitude is now reduced, as in Equation (20).

(20) $$y_{0}=\frac{2\Omega m_{C}Q_{S}x_{0}}{m_{S}\omega_{S}}$$

The sensitivity of the vibrating gyroscope upon the rotational rate Ω can be enhanced by adjusting different parameters to control the Coriolis effect. (1) Increase the driving mode amplitude $x_{0}$. (2) Increase the Coriolis mass $m_{C}$ while decreasing the sensing mass $m_{S}$. (3) Using vacuum packaging, increase the sensing quality factor $Q_{S}$.

### 2.4. Mode-Matching Effectiveness

It is often understood that matching the driving and sensing resonant frequencies improves the response of the sensing angular rate input [15,16,17]. However, it brings other problems, too. The overall system becomes very sensitive when it operates and matches the sensing resonant peak. Hence, various other parameters bring damping or an unwanted shift in the resonant frequencies.

**Figure 4:**
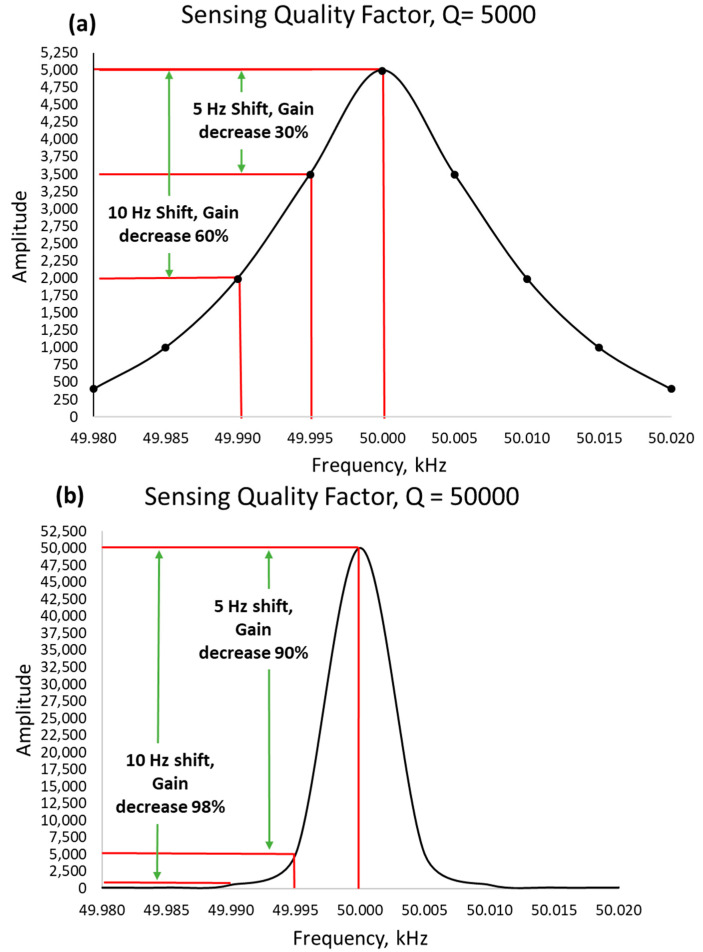
Response gain decrement of low-quality factor vs high-quality factor sensing response. (**a**) shows a response for low quality factor and (**b**) shows a response for high quality factor.

Let us suppose a gyroscopic system with a sensing resonant frequency of 50 kHz, and the sensing quality factor of about 50,000, as shown in Figure 4, is more sensitive and prone to the lost gain of 90% on just a 5 Hz frequency shift. On the other hand, a low-quality factor of about 5000 only loses 30% gain on a 5 Hz frequency shift. We can have a higher gain in high-quality factor, but the bandwidth also becomes narrower and sharp. This makes a system very sensitive to unwanted conditions. 

Many other factors contribute to the mismatch between the driving and sensing resonant modes. The shortcomings in microfabrication technologies generally affect the material characteristics and geometry [18,19]. The various deposition and etching processes affect the thicknesses and widths of the MEMS structures. These parameters change the material properties, such as Young’s modulus, affected by the deposition process [20]. The structure imbalance, temperature effect, and stresses cause a drastic shift in the operating resonant frequencies. To rectify the mode mismatch issue, it is believed to operate far from the sensing resonant frequency. The term is called frequency separation, and it is shown in Equation (21). Here, $f_{S}$ and $f_{D}$ are the sensing and driving resonant frequencies, respectively.

(21) $$\Delta f=f_{S}-f_{D}=\frac{1}{2\pi }\left(\omega_{S}-\omega_{D}\right)$$

**Figure 5:**
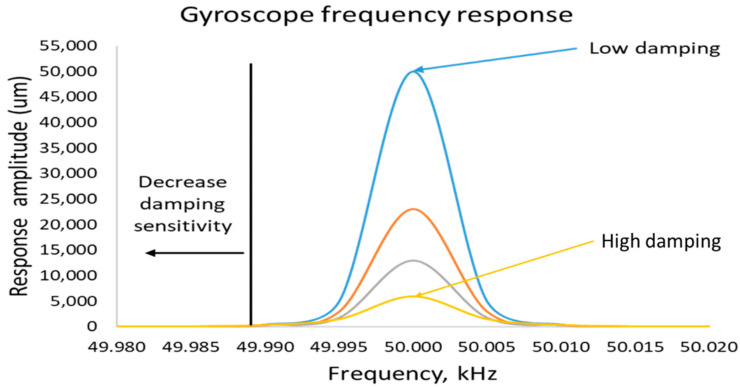
Response gain sensitivity in terms of damping effect.

The fluctuating damping parameters also contribute significantly to the mode mismatch of the resonant frequencies. It is shown in Figure 5 that low damping provides higher gain, while high damping leads to a very low response gain. The sensing quality factor $Q_{S}$ must be controlled in a very tight vacuum environment. The damping is also sensitive to temperature changes. The best way to achieve good thermal stability is to operate the system away from the resonant frequency.

## 3. Micro-Electromechanical Systems (MEMS) Vibrating Gyroscopes

In the evolution of integrated circuit (IC) technology, the miniaturization of micro-electromechanical system sensors played a significant role in scaling down the device sizes. The demand for MEMS vibrating gyroscopes in sensor applications has increased significantly because of their small and compact size, energy efficiency, overall low fabrication cost, high performance and sensitivity, and batch fabrication [21]. Exhaustive research has been conducted on many types of gyroscopes such as dual-axis, multi-axis, single-axis, single mass, dual mass, and decoupled gyroscopes. The MEMS gyroscope operates on different mechanisms. The most commonly adopted mechanisms are electrostatic [22,23] and piezoelectric [24,25], compared to electromagnet [26] and electrothermal [27] mechanisms. In recent developments, some key gyroscope designs such as vibrating-ring, tuning fork, decoupled, and dual mass [28] were found to be the most popular gyroscope structures [29]. There are four main types of MEMS vibrating gyroscopes discussed in this section.

### 3.1. Gimbal Gyroscopes 

**Figure 6:**
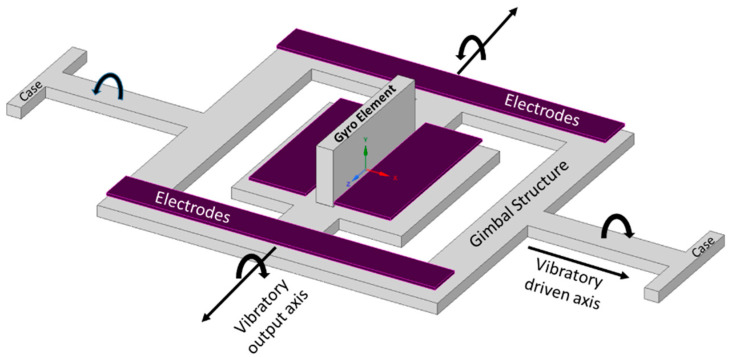
Schematic diagram of Draper laboratory’s micromachined gimbal gyroscope.

The term MEMS gimbal gyroscope is conceptualized from the traditional spinning-rotor gimbal gyroscope, where a spinning rotor is mounted on two freely gimbal systems: inner and outer gimbal systems. Earlier, in military applications, the dynamic tuning gyroscope and control moment gyroscope were used for maintaining and measuring the movement of satellites [30]. However, in the late 1980s, the Draper laboratory started its investigation on the usage of vibrating elements for gyroscopic purposes. The first micromachined gyroscope was developed by the Draper laboratory in 1988 [31]. Boxenhorn et al. demonstrated a first novel micromachined gyroscope in which there was no rotating part. The gyroscope structure was constructed on two gimbal systems: one was outer, and the other was an inner gimbal. A vertical bar was mounted on the inner gimbal. The inner gimbal constituted the sensitive element, while the outer gimbal was attached to a motor. Both gimbals were connected via orthogonal flexural pivots; the outer gimbal was used to vibrate on the given frequency and made the inner gimbal sensitive enough to detect the rotational motion on any external rotation. Furthermore, they fabricated a silicon monolithic micromechanical gyroscope. All of the design features were fabricated on the same substrate, which was the initiation of the mass production of this small-sized vibrating gyroscope. The dimensions of the gimbal structure were 350 µm × 500 µm. The outer gimbal was driven electrostatically by the electrodes at a given amplitude. This movement was transferred to the inner gimbal, making the inertial element oscillate. When rotation was applied to the gyroscope, the Coriolis force shifted the inner gimbal oscillation towards the weak axis with an equal amplitude of driving frequency. The output motion of the inner gimbal was sensed capacitively by the sensing electrodes placed near the gimbal structure. A schematic diagram of a micromachined gimbal gyroscope is shown in Figure 6 [32].

In 1996, the Draper laboratory developed the third phase of design of the vibrating wheel on the gimbal (VWOG) gyroscope. The concept had a vibrating wheel, approximately 1 mm in diameter, at a given resonance. The whole structure was suspended on a Pyrex substrate. The outer ring of the wheel structure was considered a proof mass, and there were fixed stator combs attached to the substrate and a number of combs attached to the wheel. The wheel structure had four beams suspended over the substrate and was centrally anchored to the substrate. The wheel structure was surrounded by the electrodes and those electrodes were used to excite the ring of the wheel. During operation, the wheel vibrated sinusoidally by applying a driving voltage to the fixed stator combs. When the rotation was applied to the gyroscope, the Coriolis force shifted the driving motion to the output axis. This movement was detected by the capacitor pick-off plates placed under the wheel structure and on the Pyrex substrate. The design showed excellent prospects regarding sensitivity, and the sensitivity rate was better than 0.1 deg/s in a 60 Hz bandwidth [33] in comparison with the Draper laboratory’s tuning fork gyroscope [34].

Geiger et al. demonstrated a new high-performance, low-cost, rotational rate micromachined gyroscope [35]. The concept of this gyroscope was on the mixture of the dual gimbal and comb structure gyroscope for primary and secondary oscillation. The gyroscope showed some promising test results, as the angle random walk (ARW) was measured as low as 0.14 deg/h, and the bias stability was recorded at 65 deg/h. The device structure was composed of comb drives, four fixed comb electrodes, a secondary oscillator, and the detection electrodes placed above the substrate. The comb drives were used to provide rotary motion along the *z*-axis by electrostatic actuation. The other fixed comb electrodes sensed the primary rotary motion. The Coriolis force was produced when the device rotated along the *x*-axis. The Coriolis force was used to create another rotary motion along the *y*-axis; hence, the secondary oscillator structure moved with the Coriolis force. The movement was sensed capacitively by the detection electrodes placed above the substrate.

A MEMS gyroscope was created with two gimbal structures, an inner gimbal and an outer gimbal, and was able to operate at atmospheric pressure [36]. Both the inner and outer gimbals were aligned with inner and outer coils, respectively. The whole gimbal structure was supported by torsional bars, which were placed perpendicularly to the gimbal structure. The current-carrying inner coils oscillated the inner gimbal at the given resonant frequency around the torsion bars. The outer gimbal was in a steady state because the inner gimbal oscillation was parallel to the torsion bars of the outer gimbal. The Coriolis force was generated at the center of the mass when the angular rate was applied perpendicularly. The Coriolis force was used to start oscillating the outer gimbal; subsequently, the outer coils provided an electromotive force for detecting the voltages.

**Figure 7:**
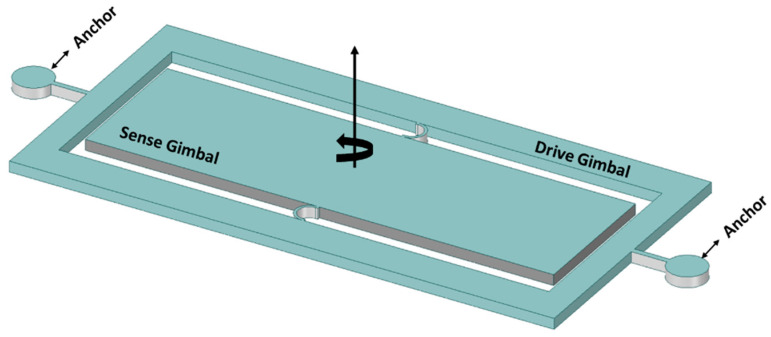
Ni–Fe alloy-based two-gimbal system gyroscope.

The two gimbal MEMS gyroscope structures containing Ni–Fe alloy were fabricated using the SU-8-based UV-LIGA process [37]. The authors claimed to overcome the shortcomings of the micromachining process using this process. Initially, a nickel layer of 8 µm was deposited as a structural layer. However, Ni material is prone to residual stresses, and the developed stresses caused the structural layer to buckle and touch the electrode that was placed beneath the device structure. This structural layer buckling issue caused the device to shorten while touching the bottom electrodes. To resolve this issue, a composition of 70% Ni, 15% Fe, and 10% C-based Ni–Fe alloy was used to avoid the buckling of the structural layer. A schematic diagram of the Ni–Fe gimbal MEMs gyroscope is shown in Figure 7.

**Figure 8:**
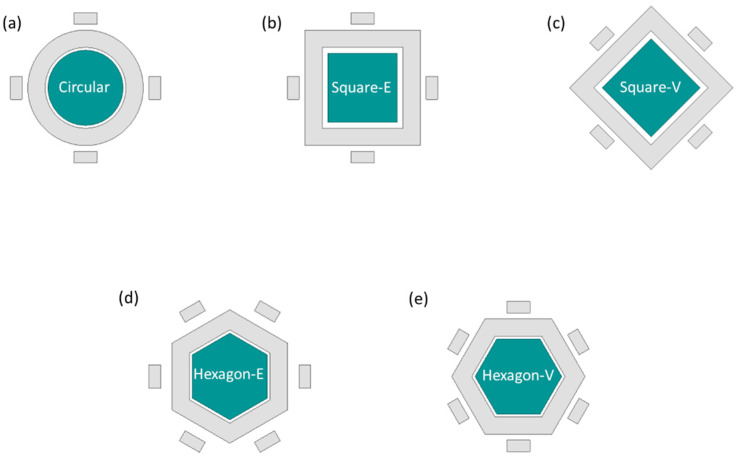
Study of different shapes of an MEMS vibrating wheel on a gimbal gyroscope. (**a**) circular (**b**) square-edge (**c**) square-vertex (**d**) hexagon-edge (**e**) hexagon-vertex.

Lee et al. investigated the scale factor and linearity error using various shapes and masses for gimbal gyroscopes [38]. Different numbers of shapes and masses were considered in this study, and the designs are shown in Figure 8. The scale factor and linearity error were extracted by the change in capacitance with various shapes of the gimbal gyroscopes. The different designs showed different results; for example, a circular-shaped structure provides a high scale factor compared to the others. However, the circular shape did not offer a good linearity error. A hexagonal-shaped structure gained the lowest linearity error and reasonable scale factor, making it a more feasible and stable design for MEMS gimbal gyroscopes.

### 3.2. Tuning Fork Gyroscopes

**Figure 9:**
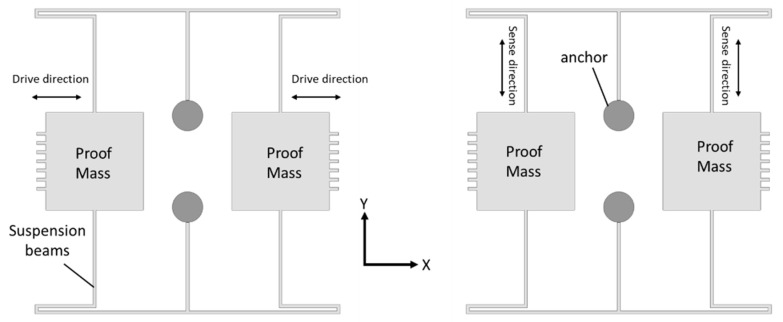
Working operation of a basic MEMS tuning fork gyroscope.

Tuning fork gyroscopes are one of the most popular designs of MEMS gyroscopes. These designs have two identical masses driven with equal amplitude, but in opposite directions [39]. When the rotational motion comes into place that is perpendicular to the driving axis, this motion produces a Coriolis force that shifts the driving motion towards the sensing axis. The driving and sensing mechanism using electrostatic actuation and capacitive sensing, respectively, provides enhanced sensitivity levels compared to the other designs [40]. A schematic illustration of the working operation of the MEMS tuning fork gyroscope is shown in Figure 9.

In 1993, the first micromachined tuning fork-rate gyroscope was developed using nickel-electroforming technology, reactive ion-etching polysilicon, and single-crystal silicon on glass technology. The size of the gyroscope was 1 mm, and the target was to achieve the bias stability of less than 100 deg/h. Initially, all three fabrication technologies were considered; however, only one was eventually selected because of its good fabrication results. Nickel-electroforming technology reduced the feature size to 6 µm, but due to the low quality and possible fatigue associated with the nickel material, it was dropped because of buckling and fatigue issues. Silicon on glass fabrication offered a very low stray capacitance, but it was limited with symmetric bond wires. Reactive ion etching with polysilicon material offered great properties such as a low corrosion rate and good thermal stability, and thus was chosen as the fabrication technology. The tuning fork gyroscope had two proof masses with perforation; both masses were driven on the given resonant frequency, but in opposite directions. When rotation is applied to the gyroscope, one proof mass goes up and the other goes down. The sensing electrodes placed beneath the proof masses capacitively sensed the change in displacement. The scale factor of 90 mV/deg/s was achieved. The resolution was 0.1 deg/s and the bias stability was 0.2 deg/s in a 1 Hz bandwidth.

Che et al. fabricated a novel tuning fork gyroscope using silicon bonding and deep reactive ion-etching (DRIE) technology [41]. The gyroscope had two symmetric proof masses with multiple driving and sensing bars. The complete design structure was supported by four anchors connected with spring beams placed on the glass substrate. The gyroscope operated electrostatically and the rotational motion was capacitively sensed at atmospheric pressure. The gyroscope was tested and a significant mode mismatch of 0.12 kHz was observed between the driving and sensing modes. The driving frequency was about 2.87 kHz, the sensing frequency was 2.99 kHz, and the quality factors were 804 and 789 for driving and sensing frequency, respectively.

**Figure 10:**
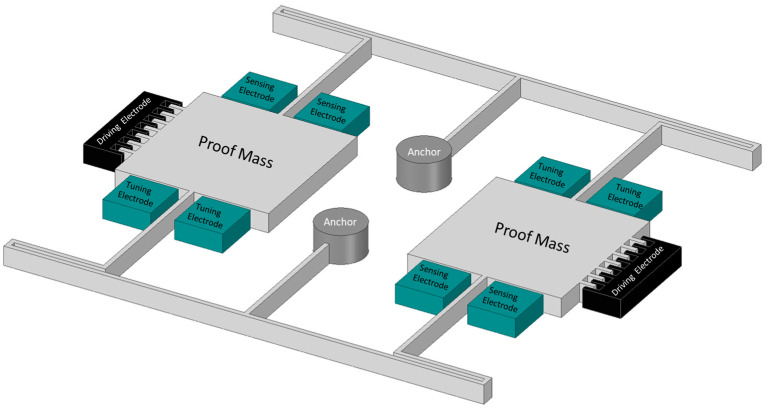
Schematic diagram of high Q MEMS tuning fork gyroscope.

A high-quality, high-resolution MEMS single-crystal silicon on an insulator tuning fork gyroscope was demonstrated in [42]. The gyroscope had two proof masses that actuate electrostatically by driving electrodes at a given resonant frequency along the *x*-axis. The Coriolis force was induced when the device experienced rotational motion around the *z*-axis, and this rotation was sensed capacitively by sensing electrodes along the *y*-axis. A schematic view of the high-quality MEMS tuning fork gyroscope is shown in Figure 10. This design achieved high-quality factors of 81,000 for the driving mode and 64,000 for the sensing mode. The gyroscope structure was fabricated on a 40 µm single silicon crystal on an insulator wafer. The simplest two-mask process was adopted for the fabrication of the gyroscope. The structural layer of moving objects and comb electrodes were first released from the backside of the wafer by etching the silicon layer using the Bosch process. Reactive ion etching was used to remove the buried oxide layer, and then the patterned layer remained to carry the suspended structure. The gyroscope achieved high resolution by electrostatically balancing the driving and sensing modes by tuning the electrodes and achieving high-quality factors with a sensitivity of 1.25 mV/deg/s.

**Figure 11:**
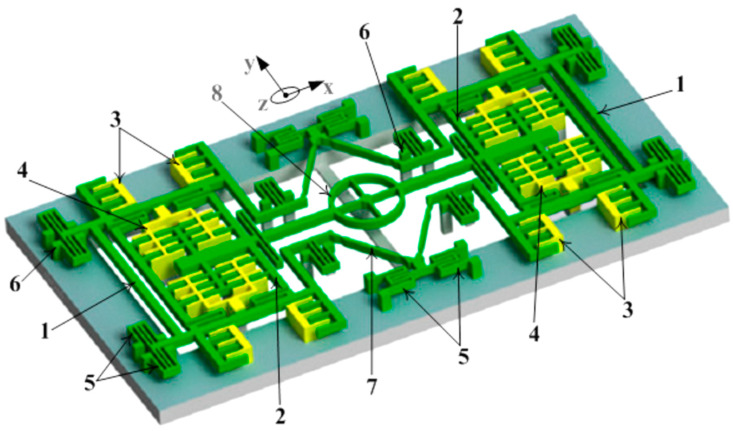
Schematic representation of the *z*-axis freestanding gyroscope [43]: 1—External frame, 2—internal frame, 3—drive electrodes, 4—sense electrode, 5—spring beams, 6—support anchors, 7—linear beams, and 8—circular rotating ring.

A *z*-axis MEMS tuning fork gyroscope that had a freestanding structure with low-air damping was developed by Nguyen et al. [43]. There were various factors considered to improve the overall performance of the gyroscope, such as eliminating the squeeze film damping by designing the driving and sensing structure to vibrate in-plane and the removal of the substrate beneath the gyroscope structure to nullify the side-film air damping. The proposed gyroscope is shown in Figure 11. The simulated driving and sensing frequencies were 9.78 kHz and 9.76 kHz, respectively. The quality factor was measured at 111.2 and the sensitivity of the gyroscope at atmospheric conditions was measured to be 11.56 mV/deg/s.

**Figure 12:**
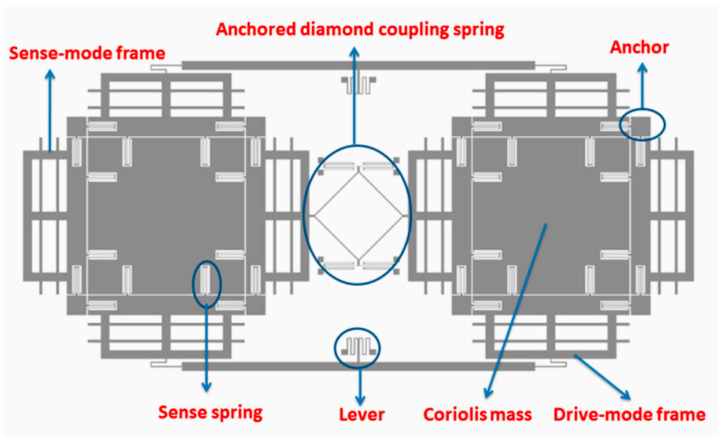
Schematic view of tuning fork gyroscope with anchored diamond coupling method [45].

Guan et al. [44] presented a paper on a new MEMS tuning fork gyroscope with anchored coupling. The new design helped to analyze the vibration mode order and the gyroscopic sensitivity. The tuning fork gyroscope utilized a levered system for drive-mode frequency and an anchored coupling spring with four beams for the sensing mode frequency. The simulated sensing frequencies recorded for the in-phase and anti-phase were 4006 kHz and 4464 kHz, respectively. However, the in-phase frequency with anchored coupling was 5958 kHz, which is almost 50% more than the simulated in-phase sensing frequency. The same researchers extended their work [45] and proposed a new design for an MEMS tuning fork gyroscope with an anchored diamond coupling method, which is shown in Figure 12. 

Trusov et al. reported a high-Q tuning fork gyroscope with a rate-integrating method, and the gyroscope demonstrated quality factors for the driving and sensing modes of 310 k and 640 k, respectively [46].

### 3.3. Vibrating Ring Gyroscopes

**Figure 13:**
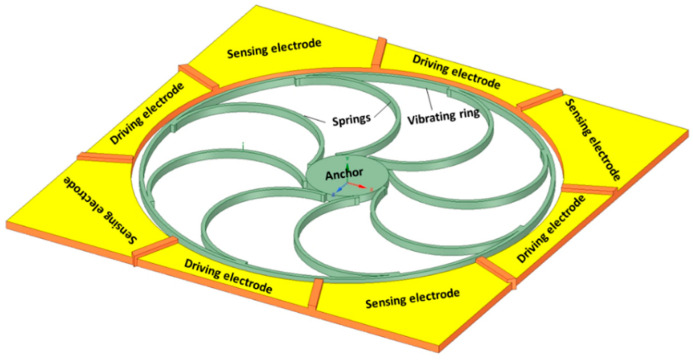
Schematic representation of the basic design of a vibrating ring gyroscope.

Vibrating ring gyroscopes have a symmetrical structure and provide many advantages over other gyroscope designs. They possess high precision, high resolution, better thermal stability, better matching of operating frequencies, a low zero-output rate, and increased sensitivity [47]. The basic design of the MEMS vibrating ring gyroscope is shown in Figure 13. It consists of an outer ring with eight springs that are supported by a circular anchor placed in the middle. 

**Figure 14:**
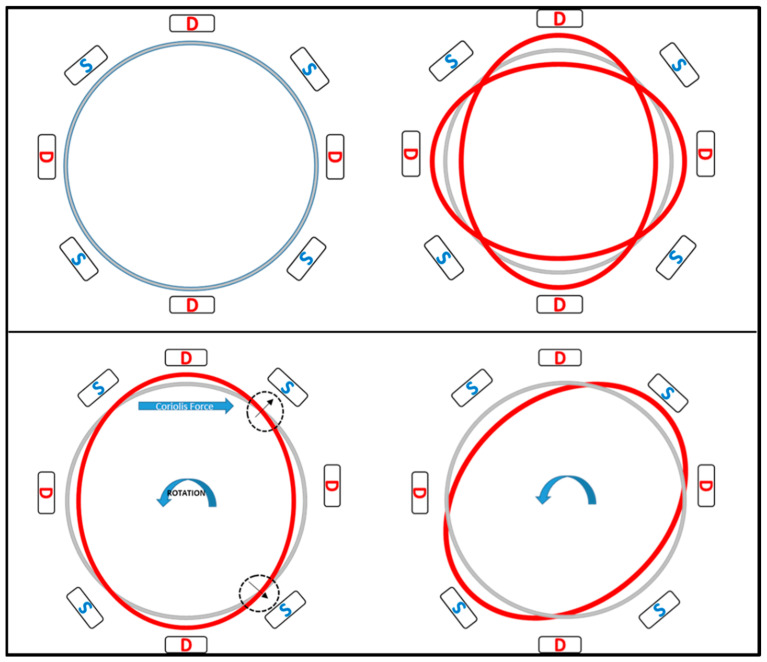
Schematic demonstration of the operation of a vibrating ring gyroscope.

The working operation of a vibrating ring gyroscope is illustrated in Figure 14. The gyroscope’s vibrating structure is surrounded by eight driving and sensing electrodes. The driving electrodes provide a continuous oscillation in the direction of the driving axis. The movement of the ring is clearly visible in the driving direction. However, there is no movement present along the sensing electrodes. When the gyroscope is exposed to external rotation, the elliptical shape of the vibration mode now transfers to the sensing electrodes. When the rotation is applied to the device, the primary vibration induces a secondary vibration mode in the sensing direction because of the generated Coriolis force. The sensing electrodes sense the change of displacement.

**Figure 15:**
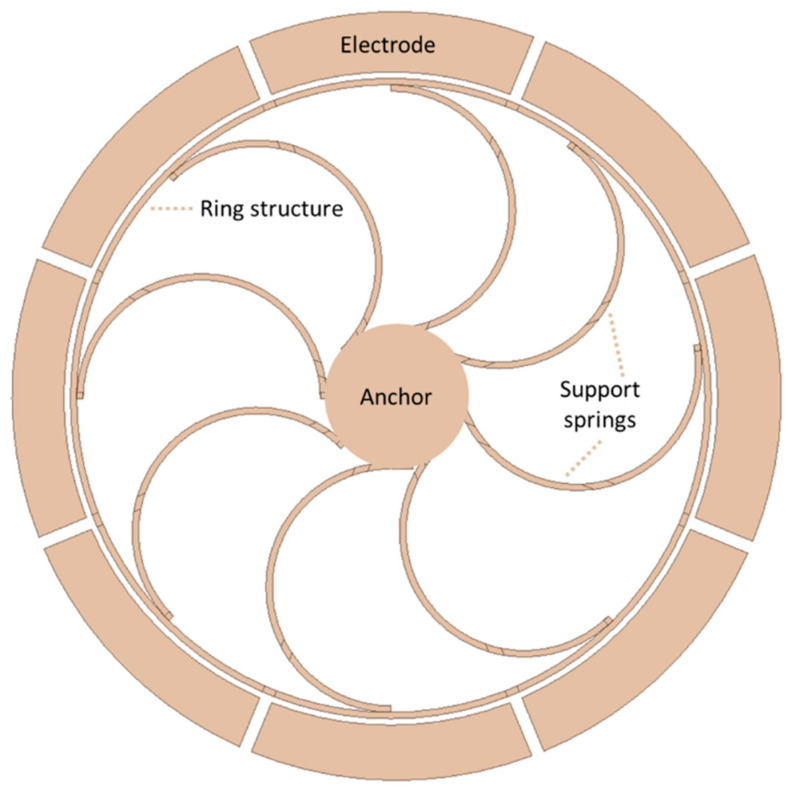
Schematic view of a novel vibrating ring gyroscope developed by General Motors.

The General Motors Corporation invented a vibrating ring gyroscope [48]. The ring structure was considered a vibrating element with high-quality radial vibrations. The ring structure was designed to be electrically conductive. There were several electrodes placed around the ring structure for the driving and sensing mechanism, as shown in the schematic diagram in Figure 15. The ring structure was supported by a centrally placed supported pillar and the ring structure was actuated by electrostatic driving electrodes. When rotation was introduced to the structure, the energy of the first vibration mode was forced to move towards 45 degrees from its position, and the sensing electrodes that were placed at 45 degrees along with the driving electrodes sensed this capacitive movement of the vibrating ring.

In 1998, a research group at the University of Michigan reported the first development of a micromachined polysilicon vibrating ring gyroscope [49]. The ring structure was 30 to 40 µm thick, while the gap between the ring and the electrode was 0.9 µm. Due to the symmetric design, the driving and sensing frequencies were almost identical, resulting in high sensitivity. The same researchers [50] presented a thorough study on vibrating ring gyroscopes for scale limits and combined microfabrication technologies such as bulk and surface micromachining. A 30 µm structural layer of the vibrating ring gyroscope was fabricated using polysilicon trench-refilling microfabrication technology, with a ring-to-electrode gap of 0.9 µm. The ARW was recorded as low as 0.05 deg/$\sqrt{h},$ and the overall sensitivity and performance level increased tremendously. There were multiple reasons for achieving the high performance of the vibrating ring gyroscope, such as polysilicon material wafer selection, less of a gap between the ring and electrode, high-quality factors, and dry-etching fabrication technology.

The same research group [51] presented a high aspect ratio polysilicon vibrating ring gyroscope fabricated using dry-release microfabrication technology. The vibrating ring gyroscope structure’s height was 80 µm. The benefit of dry-release technology was the use of a single wafer process and patterning the electrically isolated electrodes as tall as the ring structure. The sensitivity of the vibrating ring gyroscope was measured as 200 µV/deg/s within the dynamic range of ±250 deg/s under a low vacuum. The quality factor was recorded as 1200, and the driving amplitude was measured at 0.15 µm with 2 pF parasitic capacitance at the sense node.

**Figure 16:**
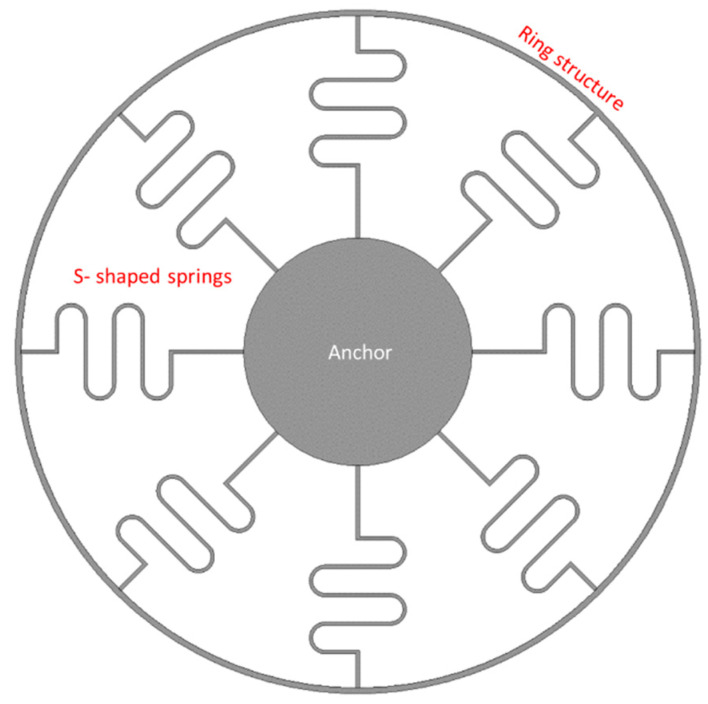
Schematic view of eight S-shaped support springs in a vibrating ring gyroscope.

A high-performance vibrating ring gyroscope was fabricated using the (111) orientation of a single-crystal silicon wafer. The vibrating ring structure of single-crystal silicon was patterned on a glass substrate with a high aspect ratio [52]. The ring radius was 1.35 mm and the thickness of the structural layer was 150 µm. The DRIE process patterned and etched a 480 µm (111) silicon wafer. The ring and electrode structure were etched to 150 µm. The wafer was further etched from the backside to release the structure layer by reactive-ion etching. The tested gyroscope achieved a quality factor of 12,000, and nonlinearity was measured at 0.02%, with higher sensitivity of 132 mv/deg/s with high resolution. The new S-shaped support springs vibrating ring gyroscope was presented by Kou et al. [53]. A ring structure was supported by eight S-shaped symmetrical support springs with centrally anchored support from the circular pillar, as shown in Figure 16. The ring structure was actuated electrostatically by driving electrodes and the rotational motion was sensed by the sensing electrodes. The whole gyroscopic structure was fabricated using a high aspect ratio microfabrication process. The gyroscope was characterized at atmospheric pressure to evaluate the overall gyroscopic performance. The operational resonant frequencies were measured at 9.844 kHz for driving and 9.865 kHz for sensing. The quality factors were 186 and 163 for driving and sensing, respectively. The zero-bias instability was recorded at 0.017 deg/s and ARW recorded 0.14 deg/$\sqrt{h}$.

Kou et al. further elaborated upon their work and carried out a further investigation on the solid-wave vibrating ring gyroscope to increase the sensitivity level [54]. A solid-wave gyroscope has many advantages, such as good reliability, wide dynamic range, and higher mechanical sensitivity. An exhaustive finite element analysis was conducted to design the gyroscope and simulate the resonant frequency and sensitivity analysis. The relationship between the design parameters and the numerical model of the sensitivity were thoroughly analyzed. The designed resonant frequency was recorded at 6.04 kHz, and the mechanical sensitivity was measured to be 0.0036 µm/deg/s.

Syed et al. [55] presented a case of design migration for an MEMS vibrating ring gyroscope from a multi-stage complex fabrication process to a simple, cost-effective process. The two different design approaches were considered for the MEMS vibrating ring gyroscope. In the first design, the multi-vibrating ring structure had C-shaped support springs connected and was also supported by the inner anchor. However, in the second design, the multi-vibrating rings with C-shaped support springs were connected and supported with the outer-placed anchor. In the first design, there were some design fabrication limitations to filling the whole area; therefore, the area was filled with more pillars. The second design provided better results for gyroscopic operation. The second design achieved better sensitivity, an easy microfabrication process, perfect wine glass vibration modes, and high shock resistibility. 

**Figure 17:**
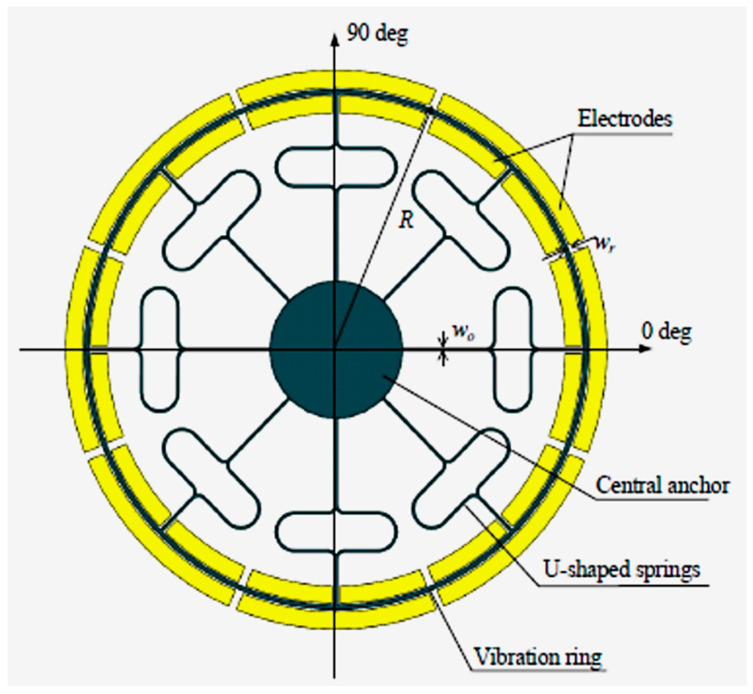
Schematic view of MEMS double U-beam vibrating ring gyroscope [56].

A double U-beam vibrating ring gyroscope was established by Cao et al. [56]. The design of the device was conceptualized with a mathematical model and finite element modeling. The gyroscope structure comprises a circular ring connected to eight double U-beam support springs, connected with a centrally placed anchor. There were twenty-four electrodes placed around the ring structure to vibrate the ring with driving electrodes and to sense changes in the capacitance due to changes in displacement by sensing electrodes. A schematic diagram of a double U-beam vibrating ring gyroscope is shown in Figure 17. The microfabrication was done with silicon on glass technology with deep reactive-ion etching. The designed, simulated resonant frequencies for driving and sensing were 9.609 kHz and 9.615 kHz, respectively. The gyroscope was tested for actual performance; the tested resonant frequencies for driving and sensing were 9.124 kHz and 9.146 kHz, respectively, which were lower in comparison to the simulated frequencies. The bias instability was measured to be 8.86 deg/h and the ARW was 0.78 deg/$\sqrt{h}$.

**Figure 18:**
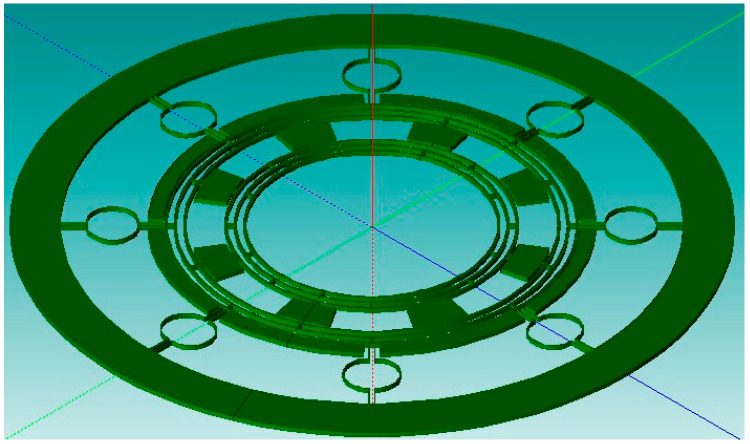
Design of a multi-ring vibrating gyroscope [57].

A novel design of a multi-ring vibrating gyroscope was established in [57]. The design had two sets of ring structures: internal rings and external rings. The multiple rings enhanced the sensitivity. The ring structure with C-shaped support springs was connected to the outer-placed fixed anchor. The proposed design of the multi-ring vibrating gyroscope is shown in Figure 18. The simulated resonant frequency was 40.40 kHz. However, when the device was characterized, the measured resonant frequency was 36.67 kHz. The difference between the designed and tested resonant values was less than 4.0 kHz. The reason for the discrepancy was microfabrication errors such as critical dimension losses and sidewall angle.

Liang et al. [58] developed a MEMS vibrating ring gyroscope that was attached to piezoelectric film. The piezoelectric film covered the circumference of the vibrating ring to adjust the rigidity of the gyroscope. The proposed piezoelectric film enhances the sensitivity because of the control of the forced oscillation and parametric resonance. The increment of the bias voltage and piezoelectric voltage can amplify the driving amplitude response of the gyroscope. Because of the forced oscillation, the sensing amplitude response is more significant due to the forced oscillation speed. When the oscillation speed is decreased, it will affect the other parameters. The best way to achieve maximum sensitivity is to design the gyroscope oscillation speed close to the optimum values. The same research group [59] further studied the nonlinearity behavior of the vibrating ring gyroscope. The findings were obtained using the nonlinear model to identify the couplings with geometric and structural coupling. It was found that cubic rigidity nonlinearity has a significant impact on the driving and sensing mode coupling.

### 3.4. Multi-Axis Gyroscopes

**Figure 19:**
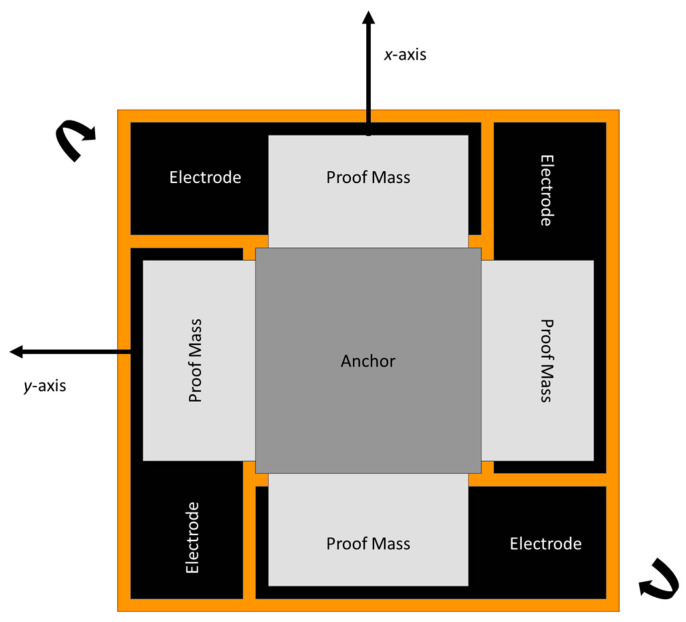
Schematic diagram of novel two-dimensional micromachined gyroscope developed by Fujita et al.

A novel two-dimensional micromechanical gyroscope was developed by Fujita et al. [60]. The novel design, which is shown in Figure 19, included four cantilever plates that were placed above the glass substrate with four fixed electrodes. When rotation was applied to the micro gyroscope, the movement was triggered by the Coriolis force and was detected capacitively upon the displacement changing between the cantilever and the fixed electrodes. They proposed to match the driving and sensing frequency for the overall enhancement of the sensitivity of the gyroscope. The sensitivity recorded for this two-dimensional gyroscope was 0.1 mV/deg/s.

**Figure 20:**
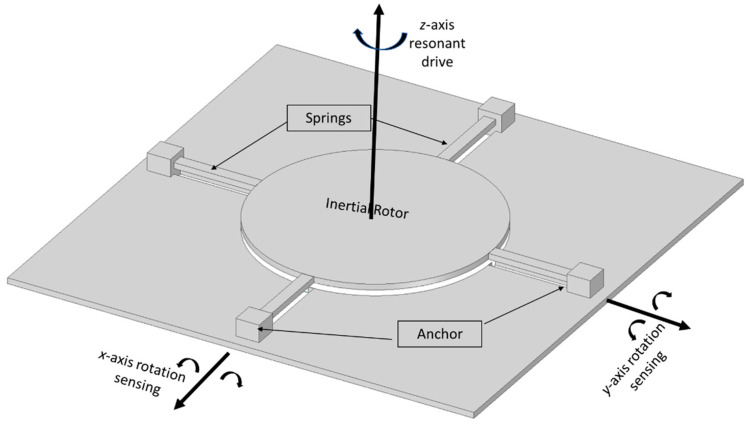
Schematic diagram of a dual-axis micromachined vibrating gyroscope.

In 1997, a dual-axis micromachined rate gyroscope was presented at the IEEE Transducers 97 Conference [61]. The gyroscope had a 2 µm thick polysilicon disk of 0.3 mm diameter placed 1.6 µm above the substrate. The disk structure was supported by four beams that were connected with four anchors to support the disk structure. The beams were designed to provide torsional suspension. The device operation had an angular resonant about the *z*-axis and the rotation on the *x*-axis produced a Coriolis response to the *y*-axis. Because of the symmetric structure of the gyroscope, any rotation on the *y*-axis produced a Coriolis response on the *x*-axis. This change was sensed capacitively by the sensing electrodes placed under the disk structure. A schematic representation is shown in Figure 20. The ARW was considerably higher due to the mismatch of the resonant frequencies and the tuning frequencies, and the ARW decreased to 2 deg/$\sqrt{h}$ from 10 deg/$\sqrt{h}$, but with high cross-axis sensitivity.

The NASA Jet Propulsion Laboratory presented its work on the fabrication, design, and packaging of the micro gyroscope for space applications [62]. The micro gyroscope had a 7 Hz split frequency between the driving and sensing mode, the scale factor was 24 mV/deg/s, the bias stability was 70 deg/h, and the ARW was 6.3 deg/$\sqrt{h}$. The whole package consisted of the micro gyroscope, preamplifier, power converter, and summation circuit. 

In 1998, a tunable micromachined gyroscope was presented by Kang et al. [63]. The device had a polysilicon structure, two suspended micro plates vibrated electrostatically by a comb structure in the antisymmetric direction, and the two electrodes were placed under the structure of the plates. When the external rotation was applied to the device, the Coriolis acceleration developed and forced the plates to move in the opposite direction or away from the bottom electrodes. This change in capacitance was sensed by the bottom electrodes.

A research group at the University of California developed a four-mass rate-integrated MEMS gyroscope. The proposed design of a four-mass gyroscope offers a dynamically balanced and highly symmetric structure. This vacuum-sealed quadruple-mass gyroscope achieved high quality factors of more than a million in driving and sensing directions. The gyroscope operated at the resonant frequency of 2 kHz. The gyroscope operated with mode matching and showed a linear response in a ±450 deg/s range and 100 Hz bandwidth [64,65]. Zotov et al. presented the first mechanical frequency-modulated angular rate gyroscope. The reported gyroscope is based on a four-mass gyroscope with a symmetrical structure on the *X*-*Y* axis. The high-range angular rate sensor was digitally tested by implementing a two-phase locked loop. The device maintained quality factors of more than a million in the driving and sensing direction, with a temperature range from −40 °C to 100 °C [66]. 

**Figure 21:**
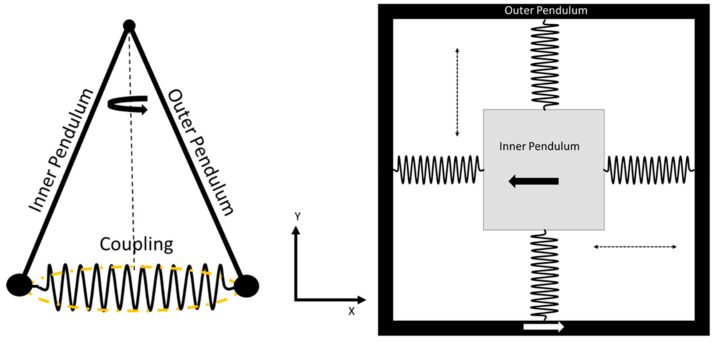
Schematic diagram of dual Foucault pendulum gyroscope. Two Foucault pendulums vibrate in antiphase motion.

Senkal et al. [28] reported a dual mass whole-angle MEMS gyroscope that was based on the dual Foucault pendulum concept. A schematic representation of the reported gyroscope is shown in Figure 21. The gyroscope has a symmetric design on the *X*-*Y* axis. The dual-axis gyroscope operated at the frequency of 2.7 kHz with more than 100 k quality factors in both driving and sensing modes. Minotti et al. [67] developed a three-axis frequency-modulated MEMS gyroscope. The yaw and pitch rates were demonstrated by coupling with two MEMS structures that were fabricated using a 24 µm structural layer process. The devices have shown a stable high scale factor. 

Table 1 presents a timeline summary of the most common MEMS vibrating gyroscopes and their development throughout the years. The Charles Stark Draper Laboratory initially started the investigation on the development of the vibrating structure for the gyroscope. Later on, many other institutions presented different design structures that provided better overall gyroscopic performances.

## 4. Reliability Issues 

The recent use of MEMS vibrating gyroscopes has risen enormously in many devices from household applications to space applications. However, the greatest challenge that needs to be addressed is the reliability of these MEMS sensors for commercialization purposes. This inertial sensor must perform accurately in extreme conditions ranging from harsh environments, corrosive environments, high temperatures, intense vibrations, and many others. These factors easily affect the overall performance of the gyroscope and raise reliability issues. To check the integrity of the gyroscope, it must go through MEMS reliability identification processes [79]. These reliability checks go through process evaluation, environmental testing, and MEMS-related failure tests. In process evaluation, the robustness of the design is tested, including the material properties, structural stability, and manufacturing process stability. These tests give an understanding of the common failure mechanisms and process stability, beam stiffness, and residual stresses on the structure. The long-term reliability check of MEMS gyroscopes requires them to be characterized in environmental testing, sometimes simulating extreme harsh environments. The environmental testing comprises various dynamic and thermal tests, such as pyro-shock, noise tests, external vibrations, thermal vacuuming, and thermal cycling tests. A pressure leak test is used for checking the integrity of the sealing of packaged gyroscopes. The third test is for investigating the failure modes and their mechanisms. The surface stiction problem is quite common and occurs at the micro-level fabrication when two surfaces that are finely polished come into contact and stick to each other. The microfabricated surfaces of the two metals are smooth and frictionless. These metal surfaces make strong bonds and due to attraction, can stick together. The other factor is external vibrations, and gyroscope sensitivity can easily decrease upon a high volume of vibrations [80]. These external vibrations can result in fatigue to the structure and cause catastrophic failure. Radiation from other sources also decreases the reliability of the gyroscope [79], where the extra reading or deviation from the desired output value or continuous developing errors are due to the radiations from other sources. High-impact radiation can create lattice imperfections that bring the structure to failure.

An investigation was carried out into the use of a gyroscope as a tracking system for fire-fighting inside buildings where GPS does not work [81]. As the extreme temperature degrades the performance of the gyroscope, the life of the firefighter could be in danger. At elevated temperatures, the minimum deviation of the scale factor can cause a high percentage of errors in the output signals. The following subsections present some key issues regarding the reliability of the MEMS gyroscope.

### 4.1. Temperature Characteristics

The performance and sensitivity of the MEMS vibrating gyroscope can significantly deteriorate at elevated temperatures. Temperature changes can have an impact on the performance; for example, an error of 1% between the driving and sensing frequency can cause a 20% error in the output signal. The role of temperature characteristics on the silicon MEMS gyroscope for a spacecraft was discussed by Shcheglov et al. [82]. The packaged silicon MEMS gyroscopes were tested at different temperatures ranging from −60 °C to 60 °C. A number of gyroscopic parameters were measured during the test, including resonant frequency, quadrature error, and bias drift. The study found that temperature fluctuations played a significant role in the low-noise drift and also affected the resonant frequency and quality factor. Temperatures above 20 °C significantly degrade the performance of the gyroscope. The recommendation was to control the environment temperature below 20 °C for better performance and enhanced gyroscope sensitivity.

A thermal cycling study was conducted on three different three-axis MEMS vibratory gyroscopes [83]. The measurements were taken for noise variation and signal output during stationary and rotational motion. The rotational test was conducted on a rotating table with multiple rotations of 60 deg/s, 120 deg/s, and 240 deg/s for five minutes each. The initial tests were performed at room temperature. Further testing was performed by thermal cycling from −25 °C to 125 °C for stationary and rotational motion testing. One process test lasted for 100 h and this procedure was repeated five times. The results confirmed the degradation and permanent deformation on the performance of the three-axis MEMS vibrating gyroscope. The stationary gyroscope tested for 500 h of the thermal cycle showed no certain change recorded for up to 400 h. However, after 400 h and up to 500 h, the degradation was observed prominently at the rate of 1 deg/s and 1.8 deg/s, respectively. The results for the rotational motion gyroscope had a degradation of 1 deg/s and 1.8 deg/s between 400 h and 500 h during the thermal cycle, respectively.

The deformation behavior of the vibrating gyroscope upon temperature changes was also investigated by Joo et al. [84]. The study was conducted in the temperature range from 0 °C to 150 °C and revealed the mismatch of the coefficient of linear expansion of the wafer cap, bonding material, and the printed circuit board upon temperature changes as the main sources of the degradation of the performance of the gyroscope. The study also found a certain frequency shift when the deformation occurred due to temperature changes. The crab-leg spring structure was introduced to provide more stability and robustness to the gyroscope package. The crab-leg spring design reduced the frequency shift by a great amount and improved the gyroscope sensitivity.

The NASA Jet Propulsion Laboratory (JPL) investigated a MEMS vibratory gyroscope for temperature dependence [85]. The experiment was conducted in the range of temperatures from 35 °C to 65 °C to find the hysteresis results. The initial results showed a strong linear relationship between the driving frequency and sensing frequency with the increase in temperature resulting in a decrease of 90 mHz/°C and the automatic gain control bias voltage with a temperature increment of 13 mV/°C. These results also indicated a significant time delay when the gyroscope was exposed to external temperature fluctuations.

Reliability is the key ingredient to the success of the commercialization of MEMS devices. The MEMS vibrating gyroscope was investigated in [86] for its reliability and long-term stability with vacuum wafer-level packaging upon various stresses at high frequencies and exposure to high temperatures. In particular, the tests were carried out for mechanical stability concerns such as vibrations, fatigue, and abnormal shocks. The quality factor number reduced significantly when the device operated at a high temperature, due to leakage and the increment of outgassing from the cavity. These leakages were the results of bonding interfacing defects between silicon wafer and glass. The ten gyroscopes were tested for drop and vibration qualification tests. They proposed a three-step method of vacuum wafer-level packaging. First, the silicon wafer needed to be prebaked in a vacuum oven. The second step was to achieve the optimum bonding interface to reduce voids and defects that give a free path to leakage. The final step was to use titanium and gold material coatings as a diffusion barrier. These three-step methods were implemented, and a number of reliability tests were carried out. The results showed the quality factor reduced very little, which proved the reliability of the new vacuum-packaging method. The gyroscope was also found safe for fatigue and high-shock environments from the drop tests. The gyroscopes were found to last 1000 h without any performance degradation.

The sensitivity stability of the gyroscope from −40 °C to 60 °C was investigated by Li et al. [87]. The mismatch between the driving and sensing frequency was adjusted by increasing the tuning voltage from 5 V to 30 V. The gyroscopic parameters resonant frequency and quality factor were characterized in the temperature range of −40 °C to 60 °C in a controlled chamber with increments of 10 °C per 30 min at each step. A small amount of discrepancy was observed for the quality factor number of the driving and sensing mode that showed the temperature difference did not decrease the sensitivity level of the gyroscope. However, the driving and sensing frequency decreased by about 10 Hz for both modes in the specified temperature range. Moreover, the mismatched frequency varied about 3 Hz in the given temperature range.

The bias instability of a gyroscope was minimized to 1 deg/h from performing about 4 deg/h in the set temperature range from −40 °C to 120 °C [88]. The stable value of 1 deg/h was achieved by the stable split resonant frequency between the driving and sensing modes. The variation in the resonant frequencies was because of the thermal stresses. The gyroscope design was modified and remained open at one side to overcome the split between the driving and sensing frequencies due to thermal stresses. The gyroscope was driven at the resonant frequency of 18.5 kHz and less than 2 Hz variation in the split resonant modes was observed. It is noticed that at 25 °C, the split between the driving and sensing mode is normalized and overall was less than 2 Hz split across the temperature range of −40 °C to 120 °C. 

Giner et al. [89] demonstrated an X-Y symmetric resonator to increase the gyroscope structure symmetry. The concept of the gyroscope was as a rate-integrating gyroscope and mechanized as a *z*-axis rate gyroscope. The design modifications were made by placing concentrated springs inside the structure of the gyroscope to overcome the fabrication errors. The design achieved a sub-Hz resonant frequency split between the driving and sensing modes. The highly symmetrical structure possessed thermal stability that corroborated its effectiveness at robust temperatures. The gyroscope had a 50 mHz split resonant frequency up to 130 °C in temperature.

In recent developments, rate-integrated gyroscopes [64,65,90] and frequency-modulated gyroscopes [66] have been established. These gyroscopes are not sensitive to temperature changes. The frequency-modulated gyroscope operates on the two oscillation modes of vibrations [91]. Rate-integrated gyroscopes are also known as whole-angle gyroscopes that operate on the antiphase oscillation concept [28]. Eminoglu et al. [92] reported a vibrating ring gyroscope based on amplitude modulation and frequency modulation. The reported gyroscope showed considerable improvements in the bias stability and scale factor. The temperature coefficients for bias decreased from 700 deg/h/°C to 3 deg/h/°C and the scale factor from 105 ppm/°C to 1.25 ppm/°C. Another frequency-modulated gyroscope demonstrated in [93] showed that the clockwise and counter-clockwise vibration modes are independently controlled on the single resonator, which compensates the temperature effect and stabilizes the scale factor and bias for over 700 h.

A Lissajous frequency-modulated gyroscope was reported with a four-mass resonator [94]. This type of gyroscope is easy to implement and does not require a complex controller like rate-integrated gyroscopes. It provided a stable response of scale factor and bias stability with removing the temperature effect. Minotti et al. [67] demonstrated a Lissajous frequency-modulated gyroscope with high scale factor stability. The same kind of three-axis Lissajous frequency-modulated gyroscope developed by Zega et al. [95] showed a relatively easy implementation in the roll/pitch axis because it does not require perfect mode matching.

A summary of the different research groups on the findings of gyroscope performance and sensitivity affected by temperature characteristics is shown in Table 2. It clearly shows that gyroscope performance significantly decreased at elevated temperatures. Gyroscope operating parameters accurately perform at room temperature. However, with the increment of temperature, the performance parameters, scale factor, and bias stability started to deviate from their initial position. To improve temperature robustness, more precise and advanced packaging processes require titanium or gold coatings, which can stop the leakage of the gases from the packaged gyroscope, and can certainly improve the sensitivity and stability of the gyroscope at elevated temperatures. 

Another class of MEMS gyroscopes, frequency-modulated gyroscopes and rate-integrated gyroscopes, demonstrated the stable responses of the scale factors and bias stability at different temperature ranges. These types of gyroscopes are not sensitive to temperature characteristics. Some of the latest research on these gyroscopes is listed in Table 3. 

### 4.2. Mode Mismatch

The performance of the MEMS vibrating gyroscopes relies on many factors. To improve the performance, some important factors to consider are: sensitivity enhancement, stability, and a reduction in the linearity errors during the readout circuit. It must minimize the mode mismatch between the two operating resonant frequencies and the driving and sensing modes to achieve high sensitivity with a high scale factor [97]. It is quite difficult to achieve the best mode matching during microfabrication processes since there are always some microfabrication processing errors because of restrictive tolerances. The sidewall angle and critical dimension losses are the most common microfabrication errors that deteriorate the performance of the gyroscope and increase the frequency split between the driving and sensing modes [98]. There are many methods in practice to minimize the mode mismatch by using prefabrication procedures, design feature variation studies, or reducing the split between the modes to 0 Hz using an automatic control system, and by tuning/controlling electrodes [99].

A *z*-axis tuning fork vibrating gyroscope with perfect mode matching was presented by Zaman et al. [100]. The gyroscope was fabricated on a 50 µm thick silicon insulator with a 0 Hz split frequency between the driving and sensing modes that contributed to higher sensitivity and greater gyroscopic performance. The mode-matched design achieved a 0 Hz split from a 2 Hz initial split. It indicated that perfect mode matching improved the sensitivity from 7.2 mV/deg/s to 24.2 mV/deg/s, and the quality factor increased from 10,000 to 40,000. The bias instability decreased to 0.96 deg/h from 5.4 deg/h with the mode-matched tuning fork gyroscope.

Chang et al. [101] demonstrated a control loop design for the mode matching of the MEMS gyroscope. The concept was to use the phase-locked loop for this design and to match the sensing frequency with the driving frequency. A phase-locked loop design comprised a voltage control oscillator, phase detection, and filter. 

A concept of an automatic mode matching using an extremum seeking controller for a single-axis MEMS vibratory gyroscope was presented in [102]. The proposed adaptive controller automatically matched the two main vibrational mode frequencies, driving, and sensing. The extremum seeking controller automatically amplified the amplitude of the sensing frequency using sensing mode stiffness. The experimental results verified the effectiveness of the automatic mode-matching adaptive controller that successfully reduced the mode mismatch value to less than 1 Hz and increased the sensitivity.

Tatar et al. [103] presented a program that simulated the effect of stress on the performance of the gyroscope. A circuit was introduced that parametrized several MEMS gyroscope models and predicted the effect of stress on the gyroscope’s zero-rate output and scale factor. The simulation results showed that 2 MPa of stress on the MEMS gyroscope developed on the order of 1.77 deg/h zero rate output and 18 deg/s of quadrature error deteriorated the sensitivity of the gyroscope. The stress level could go up to 40 MPa when temperature-induced packaging was involved. The gap between the drive and stator comb introduced a mismatch of resonant frequency between the driving and sensing modes. 

**Figure 22:**
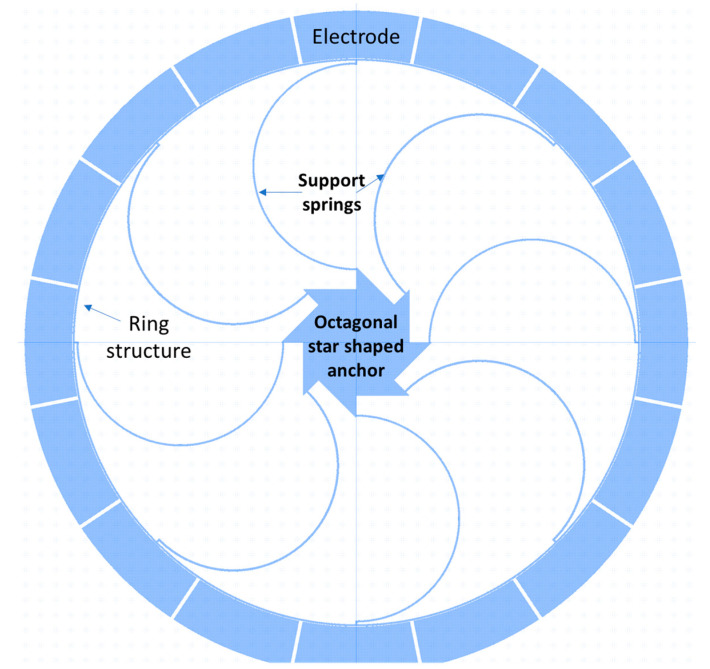
Schematic diagram of an octagonal star-shaped vibrating ring gyroscope.

An octagonal star-shaped silicon (100) vibrating ring gyroscope established by Hyun An et al. [104] compensated the length of the support springs along with <110> and <100> directions to improve the mode mismatch. A silicon (100) material has anisotropic properties in different directions. The Young’s modulus value along the direction of <110> and <100> are different, and this affected the resonant frequencies along with the driving and sensing directions [105,106]. The vibrating ring gyroscope was supported by an octagonal star-shaped anchor with eight concentrated springs, as shown in Figure 22. The parametric study of the different design features was adopted to minimize the mode mismatch without extra tuning electrodes for the silicon (100) material. The support springs with a radius length of 297 µm in the driving direction and 295 µm in the sensing direction provided a low split resonant frequency of 6 Hz from 1.184 kHz between the two resonant modes.

A vibrating disk resonator gyroscope was developed in [107] with an automatic mode-matching control system. The controller method was on the phase difference that was applied on the sensing electrodes and sensing signal to improve the scale factor and bias instability error. The proposed controller system was simulated on Simulink. This automatic mode-matching method improved the scale factor by 69 times and the bias instability by 23 times.

There are several approaches that have been presented and developed throughout recent years to achieve perfect mode matching or reduce it to a minimal value to increase the scale factor and other performance parameters. The different research institutions in the world presented phased locked-loop control systems with additional prefabrication techniques or tuned electrodes to improve the gyroscope performance. A summary of the findings of these research institutions is presented in Table 4.

## 5. Conclusions

The MEMS vibrating gyroscope is an important inertial sensor of modern-day smart electronic devices. This paper reviewed the important key fundamentals of the architecture of the sensor and its dynamic behavior. The development of the most popular designs and their different structures provide key benefits such as high scale factors and improved robustness for space and other applications. This review discussed the recent development of the gyroscope for harsh environments and highlighted reliability and stability issues at elevated temperatures and microfabrication shortcomings that degrade the performance and sensitivity of the gyroscope. Some key findings, such as prefabrication design feature processes or an automatic control loop feedback system, reduce the mode mismatch to close to 0 Hz split to enhance the gyroscopic sensitivity. Vibrating ring gyroscopes provide good thermal stability because of their design symmetry and different shapes of the support springs. They are also the best mode-matching design gyroscope because of their identical vibration modes. Dual-mass and four-mass resonator gyroscopes provide better performance at different temperature characteristics. Frequency-modulated and rate-integrated gyroscopes are not sensitive to temperature changes, and also provide stability to high scale factors with high quality factors for both driving and sensing modes. However, more research is still needed to improve the issues related to bias stability, zero-rate output, angle random walk, and others to increase the precision of these inertial sensors and to make them excellent candidates for harsh environments.

## Figures and Tables

**Table 1:** Summary of the development of the most common MEMS vibrating gyroscopes.

Design	Institution	Year	Performance Parameters	Remarks
Gimbal	The Charles Stark Draper Laboratory, USA [31]	1988	-	The first novel design of a micromachined gyroscope was established with no rotating elements. The 350 µm × 500 µm device structure was constructed with a two-gimbal system.
	The Charles Stark Draper Laboratory, USA [33]	1996	Sensitivity of 360 deg/h	A vibrating wheel on a gimbal with a given resonant suspended on a Pyrex substrate. The design shows better sensitivity than the previous designs.
	Institute of Micromachining and Information Technology, Germany [35]	1999	Sensitivity of 65 deg/h	The design comprised comb drives, comb electrodes, and primary and secondary oscillatory systems. The gyroscope sensitivity increased with the new innovative design.
	University of Hyogo, Japan [36]	2005	-	The gyroscope consisted of a two-gimbal system that can operate at atmospheric pressure.
	Khalifa University of Science and Technology, UAE [38]	2019	-	Several shapes were demonstrated for the MEMS gimbal gyroscope. A hexagonal structure provides the lowest linear error with a good scale factor.
Tuning Fork	The Charles Stark Draper Laboratory, USA [34]	1993	Sensitivity of 100 deg/h	Reactive ion-etching fabrication technique used with polysilicon material.
	Georgia Institute of Technology Atlanta, Georgia, USA [42]	2004	High quality factors of 81,000 for driving and 64,000 for sensing frequency	High-resolution single-crystal silicon on insulator gyroscope developed with higher sensitivity and higher quality factors.
	Shanghai Institute of Microsystem and Information Technology, China [41]	2009	Mismatch of 0.12 kHz with quality factors of 804 and 789 for driving and sensing frequencies, respectively	Deep reactive ion-etching fabrication technique used for this gyroscope that can operate at atmospheric pressure.
	Beijing Institute of Technology, Beijing, China [44]	2016	-	Developed a levered system for anchored coupling that increased the in-phase sensing frequency by 50%.
	Hanoi University of Science and Technology, Vietnam [43]	2017	Sensitivity of 11.56 mV/deg/s at atmospheric pressure	The proposed *z*-axis gyroscope has a freestanding structure that lowers the air damping.
	National University of Defense Technology, China [68]	2019	Bias instability of 0.59 deg/h and angle random walk of 0.04 deg/$\sqrt{h}$	A polygon shape vibration beam gyroscope with more than 100 Hz bandwidth in a scale ±200 deg/s
	Chinese Academy of Sciences, China [69]	2021	Bias instability of 9.27 deg/h and angle random walk 0.923 deg/$\sqrt{h}$	A gyroscope fabricated with 3D wafer level packaging, driving quality factor, and sensing quality factor recorded at roughly 52,000 and 49,300, respectively.
	Si-Ware Systems, Egypt [70]	2022	Bias instability of 5.5 deg/h and angle random walk 0.2 deg/$\sqrt{h}$	Roll-pitch MEMS tuning fork gyroscope developed with in-plane drive mode
Vibrating Ring	General Motors Corporation, Detroit, Michigan, USA [48]	1995	-	A vibrating ring structure was invented for a vibrating gyroscope with eight support springs.
	University of Michigan, Ann Arbor, USA [49]	1998	As low as 0.05 deg/$\sqrt{h}$ angle random walk	A first polysilicon vibrating ring gyroscope was developed with a 30 to 40 µm thick structure.
	University of Michigan, Ann Arbor, USA [52]	2002	A quality factor of 12,000 with 132 mV/deg/s	A (111) single-crystal silicon material was adopted for the gyroscope. The ring radius was 1.35 mm with 150 µm of structural layer thickness.
	University of California, Davis, USA [71]	2015	A quality factor of 80,000 with a resonant frequency of 250 kHz	A disk resonator gyroscope with a diameter of 600 µm was reported that operated at the whole-angle mode operation.
	University of California, Irvine, USA [72]	2015	A quality factor of 100 k with a stable scale factor of 20 ppm	A toroidal ring gyroscope of 1760 µm diameter fabricated with the epitaxial silicon encapsulation fabrication process.
	North University of China, China [53]	2017	Zero-bias instability measured 61.2 deg/h	A new S-shaped support spring was demonstrated for the ring gyroscope.
	Khalifa University of Science and Technology, UAE [55]	2019	-	Two different designs of multi vibrating ring structures were demonstrated to enhance the sensitivity for space applications.
	North University of China, China [56]	2019	Bias instability measured 8.86 deg/h	Double U-beam support springs were introduced to the vibrating ring gyroscope. A deep reactive ion-etching technology is used for microfabrication.
	Yangzhou University, China [58]	2020	-	The attachment of piezoelectric film increases the gyroscopic sensitivity with forced oscillation and parametric resonance.
	University of Windsor, Canada [73]	2020	Simulated resonant frequency of 64.89 kHz and experimental resonant frequency of 64.91	The rose petal-shaped support springs provided better mode matching between driving and sensing resonant modes.
	Beijing Institute of Technology, China [74]	2020	-	A hinge frame is used with the ring structure. This new design structure provides high linearity and better mode matching.
	Zhejiang University, China [19]	2021	Geometric analysis	Anisotropy of (100) single-crystal silicon affected MEMS gyroscopic properties.
Multi-axis	Himeji Institute of Technology, Japan [60]	1997	Sensitivity measured at 0.1 mV/deg/s	A novel two-dimensional design with four cantilever beams that were placed above the glass substrate.
	UC Berkeley, Berkeley, CA, USA [61]	1997	Angle random walk recorded 2 deg/$\sqrt{h}$	The gyroscope had a 2 µm thick polysilicon disk of 0.3 mm diameter placed 1.6 µm above the substrate and supported by four beams.
	Korea Advanced Institute of Science and Technology, Korea [63]	1998	-	The gyroscope had a polysilicon structure with two suspended plates that vibrated upon electrostatic actuation by comb plates.
	National Tsing Hua University, Taiwan [75]	2005	Sensitivities measured in the dual-axis sense modes, 7.4 fF/deg/s and 19.4 fF/deg/s	A novel dual-axis vibratory wheel gyroscope with three proof masses can measure the two-axis angular rate independently.
	University of California, Irvine, USA [64]	2011	Linear response in the excess of ± 450 deg/s and 100 Hz bandwidth. Driving and sensing quality factors measured 1.1 million	A four-mass MEMS vibrating gyroscope with a 2 kHz resonant frequency was developed with high quality factors.
	University of California, Irvine, USA [66]	2012	The quality factors for driving and sensing more than a million were measured in the range from −40 °C to 100 °C	A four-mass MEMS vibrating gyroscope was developed on the frequency modulation. The frequency-modulated gyroscope showed a great stable response at a different range of temperatures.
	University of California, Irvine, USA [76]	2013	A 1 ppm precision through self-calibration scale factors with temperature changes of 10 °C	A four-mass self-calibration scale factor gyroscope.
	University of California, Irvine, USA [28]	2015	100 k quality factors in driving and sensing modes at 2.7 kHz operating resonant frequency	Rate-integrating MEMS gyroscope with dual-proof masses.
	Korea University of Technology and Education, South Korea [77]	2020	-	A three-axis single-drive gyroscope was developed with a driving frequency of 25.44 kHz.
	Southeast University, China [78]	2020	Mechanical sensitivity was measured at 1.75 nm/deg/s and the micro coil sensitivity is 41.4 mOe/µm	A dual-mass MEMS gyroscope that operates by electromotive force and sensing scheme comprising differential tunneling magnetoresistance.
	Department of Mechanical and Aerospace Engineering, Politecnico di Torino, Italy [23]	2021	Minimize the cross-coupling between driving and sensing frequency by separate masses for the driving and sensing axis	A comprehensive FEA was conducted on the dual-mass gyroscope for minimizing the mode mismatch.

**Table 2:** Summary of different research group findings of gyroscope performances on temperature characteristics.

Year	Institution	Design	Temperature Range	Key Findings
2000	Jet Propulsion Laboratory, USA [82]	Silicon cloverleaf structure	−60 °C to +60 °C	Above 20 °C, the resonant frequency and quality factor decreased
2005	Jet Propulsion Laboratory, USA [85]	Post resonator structure	+35 °C to +65 °C	A decrease of 90 mHz/°C of driving and sensing frequency from the specified temperature range
2007	Chungbuk National University/Samsung Advanced Institute, China [84]	Crab-legs design	0 °C to 150 °C	Frequency shift minimized by design modification; introduced crab-leg suspension gyroscope design
2010	University of Maryland, USA [83]	Tuning fork	−25 °C to 125 °C	1 deg/s and 1.8 deg/s deviation from 400 to 500 h thermal cycle
2017	National University of Defense Technology, China [87]	Four vibrating masses with an oblique beam	−40 °C to +60 °C	Drive and sense frequency decreased 10 Hz from −40 °C to +60 °C
2017	Hitachi Research Laboratory, Japan [88]	Tuning fork	−40 °C to +120 °C	Design modification decreased the bias instability to 1 deg/h and split frequency less than 2 Hz within the temperature range
2018	Hitachi Research Laboratory, Japan [89]	Tuning fork	Up to 130 °C	50 mHz split resonant frequency between drive and sense modes up to 130 °C

**Table 3:** Study of the effect of temperature characteristics on frequency-modulated and rate-integrated gyroscopes.

Year	Institution	Design	Temperature Range	Highlights
2012	University of California, Irvine, USA [66]	Frequency-modulated	−40 °C to +100 °C	High quality factors of one million in both modes
2013	University of California, Irvine, USA [76]	Whole-angle mode	25 °C to 35 °C	Self-calibration of the scale factor over a 10 °C temperature range
2018	Tohoku University, Sendai, Japan [93]	Frequency-modulated	25 °C to 75 °C	Scale factor measured −52 ± 136 ppm/°C
2018	Dipartimento di Elettronica, Informazione e Bioingegneria, Politecnico di Milano, Milano, Italy [67]	Frequency-modulated	25 °C to 70 °C	A stable scale factor with 35 ppm/°C on the temperature range
2020	Tohoku University, Sendai, Japan [96]	Rate-integrated	20 °C to 70 °C	Achieved excellent stability of the scale factor −1.84 ± 0.62 ppm/°C

**Table 4:** Summary of different mode-matching techniques from some research institutions.

Year	Institution	Design Approach	Split Frequency	Mode-Match Frequency	Performance Parameters
2006	Seoul National University, Korea [108]	Two control loop designs for driving and sensing	72 Hz	0 Hz	Improved sensitivity from 0.8 mV/deg/s to 7.5 mV/deg/s
2008	Georgia Institute of Technology, USA [100,109]	Prefabrication design + phase-locked loop	2 Hz	0 Hz	Improved sensitivity from 7.2 mV/deg/s to 24.2 mV/deg/s
2009	University of Trento, Italy [102]	Automatic adaptive control loop feedback	200 Hz	1 Hz	Quality factor 150
2009	Georgia Institute of Technology, USA [109]	Interface architecture CMOS circuit	12 Hz	0 Hz	Sensitivity of 88 mV/deg/s and quality factor of 36,000
2011	Newcastle University, UK [110]	Displacement feedback control system	1.8 Hz	0.1 Hz	Bias stability reduced to 0.5 deg/h from 3 deg/h
2012	Middle East Technical University, Turkey [111]	Phase-related automated closed control loop system	100 Hz	20 Hz	Bias instability reduced to 0.83 deg/h from 2 deg/h
2013	Peking University, China [112]	A fuzzy algorithm-based automatic control loop system	6 Hz	0.32 Hz	Sensitivity 65.9 mV/deg/s and bias instability of 0.68 deg/s
2013	University of California, Davis, USA [113]	Phase-locked loop with proportional integral derivative controller	135 Hz	<0.5 Hz	3.29 deg/h bias instability and 60,000 quality factor
2014	Stanford University, USA [114]	Design optimization technique without tuning electrodes	>10 kHz	96 Hz	A (100) silicon material quality factor of 100 k
2014	University of Michigan, USA [115]	Whole-angle mode birdbath resonator gyroscope	10 Hz	-	Achieved a stable angular gain and resonant frequency of 10.46 kHz
2015	Southeast University, China [116]	Tuning frequency, quadrature nulling, and forced feedback automatic control loop system	32 Hz	<0.26 Hz	Sensitivity of 10.9 mV/deg/s and nonlinearity of 0.1%
2016	Stanford University, USA [117]	Differential internal electrodes design	350 Hz	0.005 Hz	4.79 deg/h of bias instability and 0.29 deg/$\sqrt{h}$ of ARW
2017	University of Cambridge, UK [118]	T-shaped anchor design modification with open loop mode matching scheme	4.7 Hz	0.98 mHz	Quality factor recorded at 1.5 million
2018	Khalifa University of Science and Technology, UAE [104]	Octagonal star-shaped anchor design modification approach	1.184 kHz	6 Hz	A (100) silicon material with 177 quality factor
2019	University of Freiburg, Germany [119]	Digital mode matching circuit on noise observations	400 Hz	7.6 Hz	Increased overall sensitivity and reduced bias instability
2020	Southeast University, China [120]	Automatic mode matching control loop with virtual Coriolis force	6.27 Hz	<0.1 Hz	Sensitivity increased from 0.226 mV/deg/s to 4.13 mV/deg/s and bias instability reduced to 2.83 deg/h from 3.76 deg/h
2021	Georgia Institute of Technology, USA [121]	Laser ablation algorithm method to minimize mode mismatch	191 Hz	12 Hz	Scale factor measured 4.12 nA/deg/s

## Data Availability

The data presented in this article is available on request from the corresponding author.

## Nomenclature

*F_D_*	driving force
*F_S_*	sensing force
*F_C_*	Coriolis force
*m*	inertial mass
*x*	displacement into driving motion
*y*	displacement into sensing motion
*c*	damping coefficient
*k*	stiffness constant
Ω	external rotation rate
$\omega_{n}$	natural frequency
ζ	damping factor
*F*_0_	initial force
$x_{0}$	initial displacement
ϕ	phase angle
$m_{D}$	driving mass
$k_{D}$	driving spring stiffness constant
$c_{D}$	driving damping constant
$m_{C}$	Coriolis mass
$\omega_{D}$	driving frequency
$m_{S}$	sensing mass
$k_{S}$	sensing spring stiffness constant
$c_{S}$	sensing damping constant
SU-8	epoxy-based negative photoresist
UV LIGA	ultra violet lithography galvanoforming aboforming
FEA	finite element analysis
